# Enveloping branes and brane-world singularities

**DOI:** 10.1140/epjc/s10052-014-3192-9

**Published:** 2014-12-12

**Authors:** Ignatios Antoniadis, Spiros Cotsakis, Ifigeneia Klaoudatou

**Affiliations:** 1 Department of Physics, CERN-Theory Division, 1211 Geneva 23, Switzerland; 2 Research Group of Geometry, Dynamical Systems and Cosmology, Department of Information and Communication Systems Engineering, University of the Aegean, Karlovassi, 83 200 Samos, Greece

## Abstract

The existence of envelopes is studied for systems of differential equations in connection with the method of asymptotic splittings which allows one to determine the singularity structure of the solutions. The result is applied to brane-worlds consisting of a 3-brane in a five-dimensional bulk, in the presence of an analog of a bulk perfect fluid parameterizing a generic class of bulk matter. We find that all flat brane solutions suffer from a finite-distance singularity contrary to previous claims. We then study the possibility of avoiding finite-distance singularities by cutting the bulk and gluing regular solutions at the position of the brane. Further imposing physical conditions such as finite Planck mass on the brane and positive energy conditions on the bulk fluid, excludes, however, this possibility as well.

## Introduction

In a previous work [1], we studied and classified the singularity structure and the corresponding asymptotic behavior of a 3-brane in a five-dimensional bulk, in the presence of an analog of a bulk perfect fluid, using the so-called method of asymptotic splittings [2, 3]. We assumed that the bulk fluid satisfies an equation of state $p=\gamma \rho$, with a constant parameter $\gamma$, while the ‘pressure’ $p$ and the ‘density’ $\rho$ are functions of the fifth coordinate $Y$. We found a surprising result that the flat brane solution does not suffer from a finite-distance singularity in the region $-1<\gamma \le -1/2$, opening the possibility of the self-tuning mechanism for the physical cosmological constant.

More precisely, this conclusion was reached as follows: for $\gamma$ outside this region, the flat brane solution found asymptotically was general, i.e. with a maximum number of arbitrary constants, and had a singularity at finite distance from the brane position. For $\gamma$ in the above region on the other hand, the singular flat brane solution had less constants and was thus considered to be particular with the general solution assumed regular having a singularity at infinity. On the other hand, as we will see later, it is possible to find the general solution for the flat brane explicitly, and it is singular for any value of $\gamma$, which created a puzzle for the meaning of the asymptotic solutions.

The solution of the puzzle is the existence of envelopes in a system of differential equations [4, 5], together with the correct interpretation of those asymptotic solutions which show a blow up at infinity. Envelopes amount to solutions with a smaller number of arbitrary constants, and they are configurations of a special nature that the asymptotic method can also pick. In the first part of this work, we study the existence and properties of envelopes and discuss their consequences in the singularity structure analysis we made in [1]. It turns out that the solution we found with the method of asymptotic splittings in the above mentioned region of $\gamma$ is not particular but a general envelope.

In the second part of this work, we study the possibility of avoiding finite-distance singularities by cutting the bulk and gluing two non-singular branches of solutions at the position of the brane [6]. We find that this is indeed possible, while the condition of finite four-dimensional Planck mass restricts the region of $\gamma$ to $-2<\gamma <-1$. We then study the possibility of having physical systems with such an equation of state by analyzing the energy conditions for a bulk perfect fluid and we find that this region is excluded.

The plan of this paper is the following: In Sect. 2, first we describe briefly the concept of envelopes; in Sect. 2.1 we give some simple examples and analyze their effect in general, while in Sect. 2.2 we set up the dynamics of our model and analyze the nature of the envelopes that it exhibits. We then compare this result with the asymptotic behaviors that we found for the same model in [1]. It follows that there always exist finite-distance singularities for all values of the parameter of the fluid, $\gamma$. Since there is no way of avoiding these singularities when the bulk space is considered as an indivisible entity, in Sect. 3, we exploit the presence of the brane that introduces a natural symmetry in the bulk and explore the possibility of avoiding finite singularities by cutting the bulk space and matching the solutions that are regular (i.e. they exhibit no finite singularities). In Sect. 3.1, we examine which range of $\gamma$ gives a finite four-dimensional Planck mass, while in Sect. 3.2, we investigate whether this range of $\gamma$ satisfies physical constraints such as the weak and strong energy conditions. In Sect. 4, we conclude and comment on questions that remain open within the framework of the class of models considered in this paper. In Appendix A, we analyze the envelopes that exist in the case of a flat or curved brane in a perfect fluid bulk for the various values of $\gamma$. Lastly, in Appendix B, we derive in detail the forms of the weak (Sect. B.1) and strong (Sect. B.2) energy conditions.

## Asymptotic behavior and existence of envelopes

In this section, we review the effect of the existence of envelopes on differential equations in general but also for the system of differential equations that describes the brane-world model we studied in [1].

First, we describe briefly the basic concepts and terminology that we use, starting with the definition of an envelope. Consider a one-parameter family of curves described by the equation

2.1 $$\begin{aligned} F(x,y,c)=0, \end{aligned}$$

where $c$ is the arbitrary parameter. An *envelope*, ${\mathcal {E}}$, is a fixed curve that is tangent to all members of this family of curves at some point. Thus, the slope of a member of the family is the same as the slope of the envelope at the point of intersection. This condition combined with the fact that the envelope itself satisfies the equation of the family (2.1) at the point of intersection leads to the following set of equations [4]:

2.2 $$\begin{aligned} F(x,y,c)=0 \quad \text {and} \quad \partial _{c} F=0. \end{aligned}$$

The equation of the envelope can be derived by elimination of the parameter $c$ in the above set of equations. It can be shown [4] that the resulting equation includes also the locus of the critical points.

This analysis can be extended to higher dimensional objects. For instance, if instead of a family of curves we have a family of surfaces given by the equation

2.3 $$\begin{aligned} f(x,y,z;c)=0, \end{aligned}$$

and there exists a surface ${\mathcal {E}}$, which is tangent to each member of this family of surfaces along a curve, then the surface ${\mathcal {E}}$ is the *enveloping surface* of (2.3). We may define the enveloping surface by elimination of $c$ in the equations

2.4 $$\begin{aligned} f(x,y,z;c)=0,\quad \partial _c f=0. \end{aligned}$$

Then it can be shown that the resulting equation consists of two in general analytically distinct groups of surfaces, one of which is the envelope of the original surface and the other is the locus of critical points, that is, the set $\nabla f=0$, cf. [5], Sect. 219.

The method described above may be applied to the general solution of a differential equation, in order to trace the envelope which is usually a solution that cannot be derived from the general solution by assigning a particular value to some arbitrary constants. Since this method may introduce other points than the envelope, a check should be performed as a last step to certify that any curve found is indeed a solution of the system of differential equations under consideration [7], p. 17–18.

### Simple examples

Here we give simple examples of differential equations to explain in general a novel relation between envelopes and asymptotic behavior traced by the method expounded in [2] and [3] which we used in our analysis of brane-world singularities in [1]. We shall show that our asymptotic method called ‘asymptotic splittings’ has the further property that if the general solution has an envelope,^1^ that is, a limiting curve or surface to which all members of the family become tangents, then it is this enveloping curve that may be picked by the method instead of the general solution. On physical and geometrical grounds, this is to be expected since the method of asymptotic splittings is concerned with the asymptotic nature of the solutions, and if a dynamical system has an envelope, then its solutions will be asymptotically tangent to it.

We first consider the equation

2.5 $$\begin{aligned} \dot{x}^2-\dot{x}t+x=0, \end{aligned}$$

which has as a general solution the one-parameter family of curves [8]

2.6 $$\begin{aligned} x=ct-c^2, \end{aligned}$$

where $c$ is a constant. Following the method of asymptotic splittings, substituting the ansatz

2.7 $$\begin{aligned} x=at^p,\quad a,p\,\,\text {constants,} \end{aligned}$$

which we call a dominant balance, we find that the equation also admits the following solution:

2.8 $$\begin{aligned} x=\frac{t^2}{4}. \end{aligned}$$

(This is also noted in [8], albeit using a different method.) This solution has no arbitrary constant (that is, it has one less than the general solution) and does not follow from the general solution (2.6). But the solution (2.8) is the envelope of (2.6), cf. [8], pp. 333-4, Fig. 8.1. Another way to see the enveloping property of (2.8) for the family (2.6), is to set $F(x,t,c)=ct-c^2-x$, and the envelope property means the simultaneous validity of the equations

2.9 $$\begin{aligned} F=0,\quad \partial _c F=0. \end{aligned}$$

Then $\partial _c F=0$ gives $t-2c=0$, or $c=t/2$, and from the first equation we find that $x=2c^2-c^2$, or $x=t^2/4$.

We therefore conclude that the dominant balance picks the envelope not the general solution, when the latter has an envelope.

A more interesting example is the equation

2.10 $$\begin{aligned} \dot{x}^2+\dot{x}x^2t+x^3=0, \end{aligned}$$

which has the general solution,

2.11 $$\begin{aligned} x=\frac{1}{ct-c^2}. \end{aligned}$$

We substitute the dominant balance (2.7) and find the non-trivial solution $a=4,p=-2$, that is,

2.12 $$\begin{aligned} x=\frac{4}{t^2}, \end{aligned}$$

and we naturally wonder whether this is the envelope of (2.11). Such an envelope, if it exists, has to satisfy the Eq. (2.9), for the function

2.13 $$\begin{aligned} F=x-\frac{1}{c(t-c)}. \end{aligned}$$

Then we calculate

2.14 $$\begin{aligned} \partial _cF=\frac{-t+2c}{(c(t-c))^2}, \end{aligned}$$

so that $c=t/2$ which putting it back in Eq. (2.11), leads to $x$ actually being given by (2.12). Thus, we find again that the dominant balance picks the envelope instead of the general solution. Note the dependence in (2.12), which is very similar to the asymptotic forms of the density we found typically in our work [1].

The condition to deduce the existence of an envelope from the first order differential equation itself is that [5], Sections 71-4, the equation $F(\dot{x},x,t)=0$ has a double root in $\dot{x}$. For example, setting $\varphi =\dot{x}$ in Eq. (2.5), we get

2.15 $$\begin{aligned} \varphi ^2-\varphi t+x=0, \end{aligned}$$

and this has a double root provided the discriminant vanishes, that is,

2.16 $$\begin{aligned} t^2-4x=0, \end{aligned}$$

which is exactly the envelope (2.8). Similarly, for (2.10) we set $\varphi =\dot{x}$ and we are led to the equation

2.17 $$\begin{aligned} \varphi ^2+\varphi x^2 t+x^3=0, \end{aligned}$$

for which the vanishing of its discriminant gives

2.18 $$\begin{aligned} (x^2t)^2-4x^3=x^3(xt^2-4)=0, \end{aligned}$$

which, excluding the trivial solution for $x$, gives precisely the envelope (2.12).

### The case of a perfect fluid bulk

In [1] we studied a model consisting of a single 3-brane embedded in a five-dimensional bulk space with metric

2.19 $$\begin{aligned} g_{5}=a^{2}(Y)g_{4}+\mathrm{d}Y^{2}, \end{aligned}$$

where $g_{4}$ is the four-dimensional flat, de Sitter or anti-de Sitter metric, and an analog of perfect fluid with an energy-momentum tensor of the form

2.20 $$\begin{aligned} T_{AB}=(\rho +p) u_{A} u_{B}-p g_{AB}, \end{aligned}$$

where $A,B=1,2,3,4,5$ and $u_{A}=(0,0,0,0,1)$ (the fifth coordinate corresponds to $Y$). We further assumed that the fluid satisfies a linear equation of state with parameter $\gamma$, i.e. $p=\gamma \rho$, where the ‘pressure’ $p$ and the ‘density’ $\rho$ are functions only of the fifth dimension, $Y$.

Following our set-up, the five-dimensional Einstein equations,

2.21 $$\begin{aligned} G_{AB}=\kappa ^{2}_{5}T_{AB}, \end{aligned}$$

can be written as

2.22 $$\begin{aligned} \frac{a'^{2}}{a^{2}}&= \frac{\kappa ^{2}_{5}}{6}\rho + \frac{k H^{2}}{a^{2}},\end{aligned}$$

2.23 $$\begin{aligned} \frac{a''}{a}&= -\kappa ^{2}_{5}\frac{(1+2\gamma )}{6}\rho , \end{aligned}$$

where the prime $(\,')$ denotes differentiation with respect to $Y$ and for brevity we write $a$ instead of $a(Y)$. The last term in (2.22) is the curvature term that allows, apart from a flat brane ($k=0$), a de Sitter ($k=1$) and anti-de Sitter brane ($k=-1$) as well. Here $H$ denotes the radius of the hyperboloid representations of de Sitter (signature $-++++$) and anti-de Sitter (signature $-\,\!-+++$) spaces as embedded in $\mathbb {R}^5$. On the other hand, the equation of energy-momentum conservation,

2.24 $$\begin{aligned} \nabla _{B}T^{AB}=0, \end{aligned}$$

becomes

2.25 $$\begin{aligned} \rho '+4(1+\gamma )\frac{a'}{a}\rho =0. \end{aligned}$$

In [1] we used a dynamical systems method, the so-called method of asymptotic splittings, to study, in a uniform way, both the flat and curved solutions. To apply this method we use the variables

2.26 $$\begin{aligned} x=a, \quad y=a', \quad w=\rho , \end{aligned}$$

and write the system (2.23), (2.25) as the following dynamical system:

2.27 $$\begin{aligned} x'&= y, \end{aligned}$$

2.28 $$\begin{aligned} y'&= -(1+2\gamma )cw x,\end{aligned}$$

2.29 $$\begin{aligned} w'&= -4(1+\gamma )\frac{y}{x}w, \end{aligned}$$

and Eq. (2.22) as

2.30 $$\begin{aligned} \frac{y^{2}}{x^{2}}=cw+\frac{k H^{2}}{x^{2}}, \end{aligned}$$

where we have set $c=2A/3$ and $A=\kappa _{5}^{2}/4$. The method of asymptotic splittings starts by identifying all possible asymptotic behaviors of the form

2.31 $$\begin{aligned} (x,y,w)=(\alpha \Upsilon ^{p},\beta \Upsilon ^{q},\delta \Upsilon ^{r}), \end{aligned}$$

where $\Upsilon =Y-Y_{s}$, $Y_{s}$ being the position of the singularity and

2.32 $$\begin{aligned} (p,q,r)\in \mathbb {Q}^{3} \quad \text {while} \quad (\alpha ,\beta ,\delta )\in \mathbb {C}^{3}{\setminus }\{\mathbf {0}\}. \end{aligned}$$

These are described in short by the dominant balances ${\mathcal {B}}=\{{\mathbf {a}},{\mathbf {p}}\}$, where ${\mathbf {a}}=(\alpha ,\beta ,\delta )$ and ${\mathbf {p}}=(p,q,r)$.

In [1] we found that for a flat brane the only possible dominant asymptotic behavior around the finite-time singularity is described by the following dominant balance:

2.33 $$\begin{aligned}&_{\gamma }{\mathcal {B}}_{1}=\left\{ \left( \alpha ,\alpha p,\frac{3}{2A}p^{2}\right) ,(p,p-1,-2)\right\} ,\nonumber \\&\quad p=\frac{1}{2(\gamma +1)},\ \gamma \ne -1/2,-1. \end{aligned}$$

To determine whether this balance corresponds to a particular or general solution we have to calculate the eigenvalues of the matrix

2.34 $$\begin{aligned} _{\gamma }{\mathcal K}_{1}=D{\mathbf {g}}({\mathbf {a}})-\text {diag}({\mathbf {p}}), \end{aligned}$$

where $D{\mathbf {g}}({\mathbf {a}})$ is the Jacobian matrix of

2.35 $$\begin{aligned} {\mathbf {g}}=\left( y,-2A\frac{(1+2\gamma )}{3}w x,-4(1+\gamma ) \frac{y}{x}w\right) ^{\intercal } \end{aligned}$$

and ${\mathbf {a}}$, ${\mathbf {p}}$ are determined by (2.33). The eigenvalues of the $_{\gamma }{\mathcal K}_{1}$ matrix constitute its spectrum, $\hbox {spec}(_{\gamma }{\mathcal K}_{1})$. The number of non-negative eigenvalues equals the number of arbitrary constants that appear in the asymptotic expansions of solutions in the form of a series defined by

2.36 $$\begin{aligned} \mathbf {x}=\Upsilon ^{{\mathbf {p}}}({\mathbf {a}}+ \Sigma _{j=1}^{\infty }\mathbf {c}_{j}\Upsilon ^{j/s}), \end{aligned}$$

where $\mathbf {x}=(x,y,w)$, $\mathbf {c}_{j}=(c_{j1},c_{j2},c_{j3})$, and $s$ is the least common multiple of the denominators of the positive eigenvalues (cf. [2, 3]). The balance $_{\gamma }{\mathcal {B}}_{1}$ corresponds thus to the general solution in a neighborhood of the singularity in our case if and only if it possesses two non-negative eigenvalues (the third arbitrary constant being the position of the singularity, $Y_{s}$). For this balance, we found [1] that

2.37 $$\begin{aligned} \hbox {spec}(_{\gamma }{\mathcal K}_{1})= \left\{ -1,0,\frac{1+2\gamma }{1+\gamma }\right\} , \end{aligned}$$

and the last eigenvalue is a function of $\gamma$, positive when either $\gamma <-1$ or $\gamma >-1/2$, and negative when $-1<\gamma <-1/2$. The cases $\gamma >-1/2$ and $\gamma <-1$ correspond to the general solution and are both characterized by the occurrence of finite-distance singularities which are of the type collapse I ($a\rightarrow 0$, $a'\rightarrow \infty$, $\rho \rightarrow \infty$), and big rip ($a\rightarrow \infty$, $a'\rightarrow -\infty$, $\rho \rightarrow \infty$), respectively, cf. [1].

On the other hand, for the range $-1<\gamma <-1/2$, the presence of a second negative eigenvalue leaves us with two choices: we may either expand in descending powers in order to meet the arbitrary constant that corresponds to the second negative eigenvalue and find in this way the expansion of the general solution at infinity,^2^ or set this arbitrary constant equal to zero and obtain the asymptotic expansion of a particular solution which is singular at finite distance. However, the particular solution obtained by setting the arbitrary constant equal to zero, as we will now show, satisfies the equation of the enveloping surface of our dynamical system and it is thus a special solution with less arbitrary constants.

Instead of looking for enveloping solutions directly from the form of the general solution, we are motivated by our considerations at the end of Sect. 2.1 [cf. Eqs. (2.15), (2.17)] to look for such solutions directly from the field equations. The only equation in which derivatives of the basic unknowns do not appear (much like the procedure mentioned previously) is the constraint. In addition, the constraint does not contain the independent variable [note that after Eq. (2.9) and also after (2.15) we eliminated the time to express everything in terms of the parameter $c$ with respect to which we looked for envelopes] but the parameter $c$. To see this clearly, we start from the constraint of the basic system, Eq. (2.30), the one-parameter family of brane ‘surfaces’

2.38 $$\begin{aligned} f(x,y,w;c)=y^2-cx^2w-kH^2=0. \end{aligned}$$

To consider the asymptotic structure of our brane cosmology for small or large values of the extra dimension $\Upsilon$, we first examine whether the family of surfaces (2.38) has an envelope. We imagine a one-parameter family of branes parameterized by $c$ and ask whether there is an *enveloping brane* to which this family asymptotes. As we show in the next section, the general solution for the case of flat branes has the same implicit form as the constraint equation (2.30). Since the initial conditions for the brane [Eq. (3.18) below] are determined by the arbitrary constants, it follows that they will depend on the parameter $c$ that defines the family of branes (2.38). It is in this indirect sense that the concept of an enveloping brane arises in the present context. We note, however, that *curved* enveloping branes may also generally exist—for the problem in question they are given in Appendix A.

For (2.38), since $\partial _c f=-x^2w$, the enveloping brane is given by the following distinct pieces:

2.39 $$\begin{aligned} \Sigma _1:\quad x=0,\quad y=\pm H\sqrt{k}, \end{aligned}$$

2.40 $$\begin{aligned} \Sigma _2:\quad y=\pm H\sqrt{k},\quad w=0. \end{aligned}$$

The enveloping brane is thus defined as the union

2.41 $$\begin{aligned} \Sigma =\Sigma _1\cup \Sigma _2. \end{aligned}$$

We note that the envelope is not an exact solution of the field equations but an asymptotic one. Here we focus on the flat case described by $_\gamma {\mathcal {B}}_1$, while the envelopes of the remaining curved cases and a special flat case valid only for $\gamma =-1/2$ are completely analyzed in Appendix A. In particular, for the range $-1<\gamma <-1/2$, for example for $\gamma =-4/5$ we find [1] the dominant balance solution

2.42 $$\begin{aligned} x&= \alpha \Upsilon ^{5/2},\end{aligned}$$

2.43 $$\begin{aligned} y&= 5\alpha /2\Upsilon ^{3/2},\end{aligned}$$

2.44 $$\begin{aligned} w&= 75/(8A)\Upsilon ^{-2}. \end{aligned}$$

For $\Upsilon \rightarrow 0$, we find that this asymptotic solution clearly approaches the enveloping brane since it satisfies the equation of $\Sigma _1$ [this is seen most clearly by solving Eq. (2.44) for $Y$ and substituting back into the other two solutions to obtain $x,y\propto w^{-5/4},w^{-3/4}$, so that for arbitrarily large $w$ we find that asymptotically $x,y\rightarrow 0$, for the flat case we are considering here]. Further, the enveloping brane is singular. We see that the asymptotic method traced this special solution instead of the general one, and we find here that this ‘last’ enveloping brane (obtained by setting the arbitrary constant corresponding to the negative eigenvalue equal to zero) is a singular limit (it will also follow from the next section that for a suitable choice of the arbitrary constants, we may obtain this solution as the envelope of the general solution that is singular).

On the other hand, the full family of solutions corresponding to the flat brane case found in [1] (i.e., Eqs. (3.84)–(3.86) in that paper) has an expansion in descending powers and is valid at infinity. This general solution blows up there in both $a$ and $a'$ while the density $\rho$ limits to zero asymptotically as $\Upsilon \rightarrow \infty$. Transferring this solution to the finite-distance position of the singularity through the transformation $\Upsilon \rightarrow 1/\Upsilon$, we see that the corresponding solution is also singular (this movable singularity is also expected to be of the same type as that at infinity, cf. [9], chap. 5). This fact leaves no room for the avoidance of finite-distance singularities, since, as we showed in [1], finite singularities exist also for $\gamma <-1$ and $\gamma >-1/2$. Taking this into account, we focus, for the rest of this paper, on re-analyzing the question of avoiding singularities by exploiting the presence of the brane, which introduces a natural symmetry in the bulk space allowing one to consider only the part of it that may be non-singular.

## Avoidance of singularities by matching solutions

In this section, we give the analytic solution of the dynamical system for the case of a flat brane and examine the possibility of avoiding singularities by cutting the bulk space and matching the solutions that are regular.

To solve the system analytically, we first substitute $k=0$ in (2.22) which gives

3.1 $$\begin{aligned} \frac{a'^{2}}{a^{2}}=c\rho . \end{aligned}$$

We then integrate (2.25) to reveal the relation between $\rho$ and $a$ which is

3.2 $$\begin{aligned} \rho =c_{1}a^{-4(\gamma +1)}, \end{aligned}$$

with $c_{1}$ an arbitrary constant. To find the solution for the warp factor $a$, we substitute (3.2) in (3.1) and then integrate. We end up with

3.3 $$\begin{aligned} a=\left( 2(\gamma +1)\left( \pm \sqrt{\dfrac{2Ac_{1}}{3}}Y+c_{2}\right) \right) ^{1/(2(\gamma +1))},\!\!\! \quad \gamma \ne -1.\nonumber \\ \end{aligned}$$

We now find the exact form of the fluid density by inserting the above solution for $a$ in (3.2), this gives

3.4 $$\begin{aligned} \rho =c_{1}\left( 2(\gamma +1)\left( \pm \sqrt{\dfrac{2Ac_{1}}{3}}Y+c_{2}\right) \right) ^{-2}. \end{aligned}$$

Substitution of our solution for $a$ and $\rho$ in (2.23) shows that the latter equation is satisfied. Our solution (3.3) and (3.4) holds for all values of $\gamma$ except from $\gamma =-1$.^3^ We therefore see that there exists a singularity at $\mp c_{2}\sqrt{3/(2A c_{1})}$ for all $\gamma \ne -1$. In particular, as $Y$ tends to $\mp c_{2}\sqrt{3/(2A c_{1})}$, $\rho$ becomes divergent irrespectively of $\gamma$ ($\gamma \ne -1$). On the other hand, the behavior of $a$ depends on $\gamma$ in the following sense: it diverges for $\gamma <-1$ and vanishes for $\gamma >-1$.

We may apply the method of finding the enveloping surface to the general solution that we have now. However, this is just equivalent to our study of the constraint in the previous section for the following reason. Solving Eq. (3.3) for $\Upsilon$ and substituting in (3.4), we see that the constant $c_2$ is eliminated and we end up exactly with Eq. (3.2) which contains only $c_1$. Therefore the constraint (3.1) [or (2.38)], which does not contain the integration constants $c_1,c_2$, can be regarded as a non-parametric (implicit) representation of the general solution. In this respect it plays a role similar to that of the $F=0$ equations, or Eqs. (2.15) and (2.17) of Sect. 2.1, with the independent variable eliminated.

From our solution (3.3)–(3.4), we see that it is possible to avoid singularities, by making an appropriate choice for the range of parameters, for example we may choose

3.5 $$\begin{aligned} \gamma <-1 \quad \text {and} \quad c_{2}\le 0, \end{aligned}$$

combined with the $+$ sign for $Y<0$ and the $-$ sign for $Y>0$. In this case, we have the solution

3.6 $$\begin{aligned} a=\left( 2(\gamma +1)\left( -\sqrt{\dfrac{2A c_{1}}{3}}|Y|+c_{2}\right) \right) ^{1/(2(\gamma +1))}, \end{aligned}$$

and

3.7 $$\begin{aligned} \rho =c_{1}\left( 2(\gamma +1)\left( -\sqrt{\dfrac{2A c_{1}}{3}}|Y|+c_{2}\right) \right) ^{-2}, \end{aligned}$$

with the brane placed at the origin $Y=0$. Clearly then both $a$ and $\rho$ are non-singular since the term $\left( -\sqrt{2A c_{1}/3}|Y|+c_{2}\right)$ is always negative. Another choice would be

3.8 $$\begin{aligned} \gamma >-1 \quad \text {and} \quad c_{2}\ge 0, \end{aligned}$$

with the $+$ sign for $Y>0$ and the $-$ sign for $Y<0$. Then we would have

3.9 $$\begin{aligned} a=\left( 2(\gamma +1)\left( \sqrt{\dfrac{2A c_{1}}{3}}|Y|+c_{2}\right) \right) ^{1/(2(\gamma +1))}, \end{aligned}$$

and

3.10 $$\begin{aligned} \rho =c_{1}\left( 2(\gamma +1)\left( \sqrt{\dfrac{2A c_{1}}{3}}|Y|+c_{2}\right) \right) ^{-2}. \end{aligned}$$

In the following section, we will show that in order to obtain a finite four-dimensional Planck mass we will have to restrict $\gamma$ in values less than $-1$. Therefore below, we analyze the solution (3.6)–(3.7), which corresponds to $\gamma <-1$.

Naturally, we assume continuity of the warp factor and energy density. We denote the value of an arbitrary constant $c_{i}$ at $Y>0$ ($Y<0$) by $c_{i}^{+}$ ($c_{i}^{-}$) and find that continuity of the warp factor at $Y=0$ leads to the condition

3.11 $$\begin{aligned} (2(\gamma +1)c_{2}^{+})^{1/(2(\gamma +1))}=(2(\gamma +1)c_{2}^{-})^{1/(2(\gamma +1))}, \end{aligned}$$

or, since $c_{2}^{+}$ and $c_{2}^{-}$ are real numbers, we have

3.12 $$\begin{aligned} c_{2}^{+}=\pm c_{2}^{-}, \end{aligned}$$

depending on the value of $\gamma$. Similarly, continuity of the density gives

3.13 $$\begin{aligned} \dfrac{c_{1}^{+}}{(c_{2}^{+})^{2}}=\dfrac{c_{1}^{-}}{(c_{2}^{-})^{2}}, \end{aligned}$$

and using (3.12) we find

3.14 $$\begin{aligned} c_{1}^{+}=c_{1}^{-}. \end{aligned}$$

On the other hand, the jump of the extrinsic curvature $K_{\alpha \beta }=1/2(\partial g_{\alpha \beta }/\partial Y)$ ($\alpha ,\beta =1,2,3,4$), is given by

3.15 $$\begin{aligned} K_{\alpha \beta }^{+}-K_{\alpha \beta }^{-}=-\kappa _{5}^{2}\left( S_{\alpha \beta }- \dfrac{1}{3}g_{\alpha \beta }S \right) , \end{aligned}$$

where the surface energy-momentum tensor $S_{\alpha \beta }$ (defined only on the brane and vanishing off the brane) is taken to be

3.16 $$\begin{aligned} S_{\alpha \beta }=-g_{\alpha \beta }f(\rho ), \end{aligned}$$

with $f(\rho )$ denoting the brane tension and $S=g^{\alpha \beta }S_{\alpha \beta }$ the trace of $S_{\alpha \beta }$. Substitution of (3.6) and (3.16) in (3.15), leads to a junction condition for the arbitrary constants

3.17 $$\begin{aligned} \sqrt{c_{1}}\left( \dfrac{1}{c_{2}^{+}}+\dfrac{1}{c_{2}^{-}}\right) = 4\sqrt{\dfrac{2 A}{3}}(\gamma +1)f(\rho (0)), \end{aligned}$$

from which we see that we have to choose the plus sign in (3.12) and then (3.17) becomes

3.18 $$\begin{aligned} \dfrac{\sqrt{c_{1}}}{c_{2}}=2\sqrt{\dfrac{2 A}{3}}(\gamma +1)f(\rho (0)). \end{aligned}$$

### Planck mass

Another condition we need to check is whether the solution we have found for the warp factor, $a$, leads to a finite four-dimensional Planck mass, for some range of the parameter $\gamma$. The value of the four-dimensional Planck mass, $M_{p}^{2}=8\pi /\kappa$, is determined by the following integral [6]:

3.19 $$\begin{aligned} \frac{\kappa _{5}^{2}}{\kappa }=\int _{-Y_{c}}^{Y_{c}}a^{2}(Y)\mathrm{d}Y. \end{aligned}$$

For our solution, Eq. (3.6), the above integral becomes,

$$\begin{aligned}&\int _{-Y_{c}}^{Y_{c}} \left( 2(\gamma +1)\left( -\sqrt{\dfrac{2 A c_{1}}{3}}|Y|+c_{2}\right) \right) ^{1/(\gamma +1)}\mathrm{d}Y\\&\quad =\dfrac{1}{2(\gamma +2)}\sqrt{\dfrac{3}{2A c_{1}}}\left( 2(\gamma +1)\left( \sqrt{\dfrac{2Ac_{1}}{3}}Y +c_{2}\right) \right) ^{(\gamma +2)/(\gamma +1)}|_{-Y_{c}}^{0}\\&\qquad -\dfrac{1}{2(\gamma +2)}\sqrt{\dfrac{3}{2A c_{1}}}\left( 2(\gamma \!+\!1)\left( \!-\sqrt{\dfrac{2Ac_{1}}{3}}Y \!+\!c_{2}\right) \right) ^{(\gamma +2)/(\gamma \!+\!1)}|_{0}^{Y_{c}}. \end{aligned}$$

In the limit $Y_{c}\rightarrow \infty$, the Planck mass remains finite only for

3.20 $$\begin{aligned} -2<\gamma <-1, \end{aligned}$$

and it takes the form

3.21 $$\begin{aligned} \frac{\kappa _{5}^{2}}{\kappa }=\sqrt{\dfrac{3}{2A c_{1}}} \dfrac{(2(\gamma +1)c_{2})^{\frac{\gamma +2}{\gamma +1}}}{ \gamma +2}. \end{aligned}$$

This result agrees with the analysis of [6] which used a particular field theory model involving a scalar field with non-trivial kinetic terms.

### Energy conditions

In the previous section, we showed that the requirement for a finite four-dimensional Planck mass restricts $\gamma$ in the interval $(-2,-1)$. Here we discuss whether this range of $\gamma$ is allowed or somehow prohibited by physical constraints such as the energy positivity conditions. To answer this question, we first construct the weak and strong energy conditions for our type of matter (2.20) and then examine for which ranges of $\gamma$ they hold true.

We note that our metric (2.19) and our fluid are static with respect to $t$. We may reinterpret our fluid as an anisotropic one having the following energy-momentum tensor:

3.22 $$\begin{aligned} T_{AB}^\mathrm{{new}}&= (\rho ^\mathrm{{new}}+ p^\mathrm{{new}})u_{A}^\mathrm{{new}}u_{B}^\mathrm{{new}} +p^\mathrm{{new}}g_{\alpha \beta }\delta _{A}^{\alpha }\delta _{B}^{\beta }\nonumber \\&+ p_{Y}g_{55}\delta _{A}^{5}\delta _{B}^{5}, \end{aligned}$$

where $u_{A}^\mathrm{{new}}=(a(Y),0,0,0,0)$, $A,B=1,2,3,4,5$, and $\alpha ,\beta =1,2,3,4$. When we combine (2.20) with (3.22) we get the following set of relations:

3.23 $$\begin{aligned}&p_{Y}=\rho \end{aligned}$$

3.24 $$\begin{aligned}&\rho ^\mathrm{{new}}=p\end{aligned}$$

3.25 $$\begin{aligned}&p^\mathrm{{new}}=-p. \end{aligned}$$

The last two relations imply that

3.26 $$\begin{aligned} p^\mathrm{{new}}=-\rho ^\mathrm{{new}}, \end{aligned}$$

which means that this type of matter satisfies a cosmological constant-like equation of state. Imposing further $p=\gamma \rho$ and using (3.23) leads to

3.27 $$\begin{aligned} p_{Y}=\frac{p}{\gamma }. \end{aligned}$$

Substituting (3.25), (3.26), and (3.27) in (3.22), we find that

3.28 $$\begin{aligned} T_{AB}^\mathrm{{new}}= -p g_{\alpha \beta }\delta _{A}^{\alpha }\delta _{B}^{\beta }+ \frac{p}{\gamma }g_{55}\delta _{A}^{5}\delta _{B}^{5}. \end{aligned}$$

We are now ready to form the energy conditions for our type of matter. We begin with the weak energy condition according to which, every future-directed timelike vector $v^{A}$ should satisfy

3.29 $$\begin{aligned} T_{AB}v^{A}v^{B}\ge 0. \end{aligned}$$

This condition implies that the energy density should be non negative for all forms of physical matter [10]. Here we find (see Appendix B, Sect. B.1) that it translates to

3.30 $$\begin{aligned} p\ge 0 \end{aligned}$$

and

3.31 $$\begin{aligned} \gamma >0, \quad \text {or},\quad \gamma <-1. \end{aligned}$$

On the other hand, we have to exclude the case $\gamma <-1$ since when it is combined with (3.30) it gives a negative $\rho$ which is in contradiction with (3.1). We end up with the condition

3.32 $$\begin{aligned} p\ge 0 \quad \text {and}\quad \gamma >0. \end{aligned}$$

The strong energy condition, on the other hand, demands that

3.33 $$\begin{aligned} \left( T_{AB}-\dfrac{1}{3}T g_{AB}\right) v^{A}v^{B}\ge 0, \end{aligned}$$

for every future-directed timelike vector $v^{A}$. In our case, this condition leads to the following restrictions for $p$ and $\gamma$ (see Sect. B.1): We should either have $p=0$ or

3.34 $$\begin{aligned} p< 0 \quad \text {and}\quad -1\le \gamma <0, \end{aligned}$$

or,

3.35 $$\begin{aligned} p> 0 \quad \text {and}\quad 0<\gamma \le 1. \end{aligned}$$

The conditions (3.32), (3.34), and (3.35) show that the weak and strong energy conditions restrict $\gamma$ to be, in either case, greater than $-1$. This means that the type of matter considered in this paper, cannot at the same time satisfy the energy conditions *and* the requirement for a finite Planck mass given by (3.20).

## Conclusions

In the first part of this paper, we studied the effect that the existence of envelopes brings into the dynamics of the cosmological model of [1], consisting of a 3-brane embedded in a five-dimensional fluid bulk satisfying an analog of an equation of state $p=\gamma \rho$. The fluid leads to the appearance of singularities within finite distance for $\gamma <-1$ and $\gamma >-1/2$, [1]. For $-1<\gamma <-1/2$, we showed presently that singular behavior is dictated by both a particular solution that cannot be assumed as a less general behavior since it belongs to the singular part of the enveloping surface of the dynamical system in question (enveloping brane), and also by the fact that the general asymptotic solution at infinity when transformed to the finite-distance position is also singular.

The fact that the general solution as well as the enveloping branes are both singular for all values of $\gamma$ once the whole indivisible bulk is considered, led us to the second part of this paper, in which we examined the possibility of avoiding the singularities by cutting the bulk space and matching the solutions that correspond to its regular part at the position of the brane. The regular solutions that we obtain in this way, give a finite four-dimensional Planck mass for $-2<\gamma <-1$. The next question is if this range can be realized by a physical system. For this we examined an analog of the weak and strong energy conditions for the five-dimensional fluid and found that the above region of $\gamma$ does not satisfy them. This seems to be consistent with the result of [6] that used a field theory model realizing such an equation of state and found a tachyonic instability.

It is in principle possible that a singular general solution may possess an envelope having a regular piece. Unfortunately, as we showed in this work, this is not the case with the family of models considered in the present paper. It is an open question whether the form we used for the weak and strong energy conditions for the bulk system can be avoided (for example, by considering other canonical reductions), or whether a more general equation of state can be realized by considering for instance $\gamma$ non-constant but a function of the fifth coordinate $Y$. This would probably require analyzing a sort of interacting mixture on the bulk.

## Appendix A: Envelopes

In this section, we list the dominant balances that we found for the case of a perfect fluid bulk in [1] and find the part of the enveloping surface to which they belong, that is, we give the explicit structure of the enveloping branes.

The dominant balances are

A.1 $$\begin{aligned} {}_{\gamma }{\mathcal {B}}_{1}&= \left\{ \left( \alpha ,\alpha p,\frac{3}{2A}p^{2}\right) ,(p,p-1,-2)\right\} ,\nonumber \\&p=\frac{1}{2(\gamma +1)}, \, \gamma \ne -1/2,-1, \end{aligned}$$

A.2 $$\begin{aligned} _{\gamma }{\mathcal {B}}_{2}=\{(\alpha ,\alpha ,0),(1,0,-2)\},\quad \gamma \ne -1/2, \end{aligned}$$

A.3 $$\begin{aligned} _{-1/2}{\mathcal {B}}_{3}=\{(\alpha ,\alpha ,0),(1,0,r)\}, \end{aligned}$$

A.4 $$\begin{aligned} _{-1/2}{\mathcal {B}}_{4} =\{(\alpha ,\alpha ,\delta ),(1,0,-2)\}, \end{aligned}$$

A.5 $$\begin{aligned} _{-1/2}{\mathcal {B}}_{5}=\{(\alpha ,0,0), (0,-1,r)\}, \end{aligned}$$

where $$_{-1/2}{\mathcal {B}}_{i}\equiv _{\gamma =-1/2}{\mathcal {B}}_{i}$$.

The first balance, $_{\gamma }{\mathcal {B}}_{1}$, satisfies the constraint equation, Eq. (2.30), for $k=0$ immediately, that is, in the first step of the method of asymptotic splittings, where $j=0$ in Eq. (2.36). This does not imply, however, that it cannot also describe a solution for a curved brane. In fact, for this balance the curvature term, $k H^{2}/a^{2}$, has a subleading behavior that can contribute later on in the series expansion, in the way that it sets one of the arbitrary constants equal to a specific value. For example, for $\gamma =0$ the curvature term contributes at the second step of the asymptotic method, that is, at $j=1$, and sets the arbitrary constant $c_{1\,3}$ equal to $-3kH^{2}/(4A \alpha ^{2})$. We therefore see that for $k=0$, the arbitrary constant $c_{1\,3}$ is vanishing which means that the balance $_{\gamma }{\mathcal {B}}_{1}$ constitutes the general solution of the system for a flat brane. For a curved brane, on the other hand, one needs to go further up the series expansion (one more step) to find the asymptotic form of the general solution. A similar behavior is found for the balance $_{-1/2}{\mathcal {B}}_{5}$. In the latter case, the curvature term sets the arbitrary constant $c_{11}^{2}$ equal to $k H^{2}$ at $j=2$. The next two balances, $_{\gamma }{\mathcal {B}}_{2}$ and $_{-1/2}{\mathcal {B}}_{3}$, satisfy the constraint equation only when $\alpha ^{2}=kH^{2}$ and describe therefore solutions of curved branes. Finally, for the balance $_{-1/2}{\mathcal {B}}_{4}$, we find that it describes a solution for a curved or flat brane with $\delta =(3/(2A))(1-kH^{2}/\alpha ^{2})$.

The second balance, $_\gamma {\mathcal {B}}_2$, for $\gamma <-1/2$, for example $\gamma =-3/4$, gives

A.6 $$\begin{aligned} x= \alpha \Upsilon +\frac{A\alpha }{6}c_{1\,3}\Upsilon ^{2}+\cdots , \end{aligned}$$

A.7 $$\begin{aligned} y= \alpha +\frac{A\alpha }{3} c_{1\,3}\Upsilon +\cdots , \end{aligned}$$

A.8 $$\begin{aligned} w= c_{1\,3}\Upsilon ^{-1}+\cdots , \end{aligned}$$

where $c_{1\,3}$ is an arbitrary constant such that $c_{1\,3}\ne 0$. This is on $\Sigma _1$, Eq. (2.39), since as $\Upsilon \rightarrow 0$, it approaches this part of the envelope. The same balance for $\gamma >-1/2$, for example $\gamma =0$, gives

A.9 $$\begin{aligned} x= \alpha \Upsilon +c_{-1\,1}-A\alpha /3c_{-2\,3}\Upsilon ^{-1}+\cdots , \end{aligned}$$

A.10 $$\begin{aligned} y= \alpha +A\alpha /3 c_{-2\,3}\Upsilon ^{-2}+\cdots , \end{aligned}$$

A.11 $$\begin{aligned} w= c_{-2\,3}\Upsilon ^{-4}+\cdots , \end{aligned}$$

where $c_{-1\,1}$ and $c_{-2\,3}$ are arbitrary constants. For $\Upsilon \rightarrow \infty$ we see that this is on $\Sigma _2$.

The balance $_{-1/2}{\mathcal {B}}_3$ for $r=-3$ implies the following asymptotic behavior:

A.12 $$\begin{aligned} x= \alpha \Upsilon +\cdots , \end{aligned}$$

A.13 $$\begin{aligned} y= \alpha +\cdots , \end{aligned}$$

A.14 $$\begin{aligned} w= c_{1\,3}\Upsilon ^{-2}+\cdots , \end{aligned}$$

with $c_{1\,3}$ an arbitrary constant. Taking $\Upsilon \rightarrow 0$, shows that this is on $\Sigma _1$. For the same balance but for $r=0$ we have

A.15 $$\begin{aligned} x= \alpha \Upsilon +c_{-1\,1}, \end{aligned}$$

A.16 $$\begin{aligned} y= \alpha , \end{aligned}$$

A.17 $$\begin{aligned} w= c_{-2\,3}\Upsilon ^{-2}+\cdots , \end{aligned}$$

where $c_{-1\,1}$ and $c_{-2\,3}$ are arbitrary constants. Taking $\Upsilon \rightarrow \infty$ demonstrates that this is on $\Sigma _2$.

Lastly, for the balance $_{-1/2}{\mathcal {B}}_5$ and necessarily for $r=1$ (see [1]) we find

A.18 $$\begin{aligned} x= \alpha +c_{1\,1}\Upsilon +\cdots , \end{aligned}$$

A.19 $$\begin{aligned} y= c_{1\,1}+\cdots , \end{aligned}$$

A.20 $$\begin{aligned} w= 0+\cdots . \end{aligned}$$

Taking $\Upsilon \rightarrow 0$, we see that this asymptotic solution belongs to the piece $\Sigma _2$, Eq. (2.40) of the enveloping surface for both flat and curved branes since the arbitrary constant $c_{1\,1}$ in Eq. (A.19) is set to the value $c_{11}^{2}=kH^{2}$ by the constraint equation, as mentioned in the beginning of this section.

## Appendix B: Energy conditions

In this appendix, we form the weak and strong energy conditions for the type of fluid considered in this paper by adopting the formalism expounded in [11] p. 28–31 for our model. Since our medium is clearly anisotropic, it is not possible to make statements similar to the situation in general relativity. We assume that the energy-momentum tensor given by Eq. (3.22) can be decomposed in the following way:

B.1 $$\begin{aligned} \hat{T}^{AB}&= \rho ^\mathrm{{new}}\hat{e_{1}}^{A}\hat{e_{1}}^{B}+ p^\mathrm{{new}}\hat{e_{2}}^{A}\hat{e_{2}}^{B}+ p^\mathrm{{new}}\hat{e_{3}}^{A}\hat{e_{3}}^{B}\nonumber \\&+ p^\mathrm{{new}}\hat{e_{4}}^{A}\hat{e_{4}}^{B}+ p_{Y}\hat{e_{5}}^{A}\hat{e_{5}}^{B}, \end{aligned}$$

where the vectors $\hat{e_{M}}^{A}$ consist an orthonormal basis and

B.2 $$\begin{aligned} g_{AB}\hat{e_{M}}^{A}\hat{e_{N}}^{B}=\eta _{M N}, \end{aligned}$$

$\eta _{M N}$ being the Minkowski metric. Inserting the expressions of $p_{Y}$, $\rho ^\mathrm{{new}}$ and $p^\mathrm{{new}}$, given by Eqs. (3.23)–(3.25), in (B.1) we find

B.3 $$\begin{aligned} \hat{T}^{AB}&= p\hat{e_{1}}^{A}\hat{e_{1}}^{B}- p\hat{e_{2}}^{A}\hat{e_{2}}^{B}- p\hat{e_{3}}^{A}\hat{e_{3}}^{B}\nonumber \\&- p\hat{e_{4}}^{A}\hat{e_{4}}^{B}+ \dfrac{p}{\gamma }\hat{e_{5}}^{A}\hat{e_{5}}^{B} \end{aligned}$$

To set up the energy conditions we will need a normalized future-directed timelike vector $v^{A}$ which may be decomposed in the following way:

B.4 $$\begin{aligned} v^{A}&= w(\hat{e_{1}}^{A}+a \hat{e_{2}}^{A}+b \hat{e_{3}}^{A}+c \hat{e_{4}}^{A}+d \hat{e_{5}}^{A}),\nonumber \\&w=(1-a^{2}-b^{2}-c^{2}-d^{2})^{-1/2}, \end{aligned}$$

where $a,b,c,d$ are such that

B.5 $$\begin{aligned} a^{2}+b^{2}+c^{2}+d^{2}<1. \end{aligned}$$

In the following, we derive the energy conditions the final forms of which were used in Sect. 3.2.

### B.1 Weak energy condition

For any timelike vector $t^A$, we assume that $T_{AB}t^At^B\ge 0$, and so the energy density measured by an observer having velocity $v^{A}$, which is represented by the quantity $T_{AB}v^{A}v^{B}$, must be non-negative, i.e.,

B.6 $$\begin{aligned} T_{AB}v^{A}v^{B}\ge 0. \end{aligned}$$

Substituting $\hat{T}_{AB}$ and $v^{A}$ in the above relation we get

B.7 $$\begin{aligned} p-p a^{2}-p b^{2}-p c^{2}+\dfrac{p}{\gamma }d^{2}\ge 0. \end{aligned}$$

Since $a,b,c,d$ are arbitrary, we may choose $a=b=c=d=0$ (and similarly $a=c=d=0$ or $a=b=d=0$) and find that

B.8 $$\begin{aligned} p\ge 0. \end{aligned}$$

On the other hand, setting $a=b=c=0$ gives

B.9 $$\begin{aligned} p+\dfrac{p}{\gamma }d^{2}\ge 0. \end{aligned}$$

Because of (B.5), we must have $d^{2}<1$ which leads to

B.10 $$\begin{aligned} \dfrac{\gamma +1}{\gamma }p>0. \end{aligned}$$

In combination with (B.8), we find two possible ranges for $\gamma$:

B.11 $$\begin{aligned} \gamma >0, \quad \text {or},\quad \gamma <-1. \end{aligned}$$

The second range of $\gamma$ is excluded, since it implies

B.12 $$\begin{aligned} \rho =\dfrac{p}{\gamma }<0 \end{aligned}$$

which contradicts Eq. (3.1). Therefore, the weak energy condition restricts $p$ and $\gamma$ in the following way:

B.13 $$\begin{aligned} p\ge 0 \quad \text {and} \quad \gamma >0. \end{aligned}$$

### B.2 Strong energy condition

The strong energy condition states that

B.14 $$\begin{aligned} \left( T_{AB}-\dfrac{1}{3}T g_{AB}\right) v^{A}v^{B}\ge 0, \end{aligned}$$

where $T$ is the trace of $T_{AB}$. In our case,

B.15 $$\begin{aligned} \hat{T}=-4p+\dfrac{p}{\gamma }. \end{aligned}$$

Inserting in the general form of the strong energy condition given by (B.14), the Eqs. (B.4), (B.15) and $\hat{T}_{A B}$ we find

B.16 $$\begin{aligned} w^{2}\left( p-p a^{2}-p b^{2}-p c^{2} +\dfrac{p}{\gamma }d^{2}\right) \ge -\dfrac{1}{3}\left( -4p+\dfrac{p}{\gamma }\right) .\!\!\nonumber \\ \end{aligned}$$

For $a=b=c=d=0$, Eq. (B.4) gives $w=1$ and the above relation becomes

B.17 $$\begin{aligned} \dfrac{\gamma -1}{\gamma } p\le 0. \end{aligned}$$

Putting in turn, $a=c=d=0$, $a=b=d=0$, or, $b=c=d=0$, leads to the same result. On the other hand, taking $a=b=c=0$, gives

B.18 $$\begin{aligned} \dfrac{1}{1-d^{2}}\left( p+\dfrac{p}{\gamma }d^{2}\right) \ge \dfrac{1}{3}\left( 4p-\dfrac{p}{\gamma }\right) , \end{aligned}$$

or,

B.19 $$\begin{aligned} -p+\dfrac{p}{\gamma }\ge -d^{2}\left( 4p+2\dfrac{p}{\gamma }\right) \end{aligned}$$

and using the fact that $d^{2}<1$, we end up with

B.20 $$\begin{aligned} \dfrac{\gamma +1}{\gamma }p\ge 0. \end{aligned}$$

Combining Eqs. (B.17) and (B.20) we see that we have either $p=0$ or

B.21 $$\begin{aligned}&\!\!\!p< 0 \quad \text {and} \quad -1\le \gamma <0,\quad \text {or},\end{aligned}$$

B.22 $$\begin{aligned}&\!\!\!p> 0 \quad \text {and} \quad 0<\gamma \le 1. \end{aligned}$$

## Footnotes

1 In general, any system of dynamical equations may itself define an envelope (for instance, when one considers a differential system with constraints) irrespective of whether or not the general solution is known. A general theory connecting the method of asymptotic splittings with the existence of envelopes for a general differential dynamical system is at present unknown.

2 In [1], the solution obtained for $\gamma =-4/5$, Eqs. (3.84)–(3.86) in that paper, was wrongly termed ‘particular’, it is a general solution of the brane equations. The correct eigenvector is the one that corresponds to the eigenvalue $-3$ of the transpose matrix of $_{-4/5}{\mathcal K}_{1}$. This eigenvector is $(75/(4 A\alpha ),-15/(2A \alpha ),1)$ and then the compatibility condition at $j=-3$ is satisfied. Therefore we do not have to set $c_{-1\,1}=0$ and the solution for $\gamma =-4/5$ is indeed general.

3 We may solve the system (3.1), (2.23), and (2.25) for $\gamma =-1$ in order to have a complete picture of the dynamics. Inserting $\gamma =-1$ we find that $\rho$ is a constant and $a$ has an exponential form which means that there are no finite-distance singularities in this case. This behavior is anticipated since $\gamma =-1$ corresponds to a cosmological constant.
